# Exploring the Solar Wind‐Planetary Interaction at Mars: Implication for Magnetic Reconnection

**DOI:** 10.1029/2022JA030989

**Published:** 2023-02-10

**Authors:** Charles F. Bowers, Gina A. DiBraccio, James A. Slavin, Jacob R. Gruesbeck, Tristan Weber, Shaosui Xu, Norberto Romanelli, Yuki Harada

**Affiliations:** ^1^ Department of Climate and Space Sciences and Engineering University of Michigan Ann Arbor MI USA; ^2^ Solar System Exploration Division NASA Goddard Space Flight Center Greenbelt MD USA; ^3^ Department of Physics and Astronomy Howard University Washington DC USA; ^4^ Space Sciences Laboratory University of California Berkeley CA USA; ^5^ Department of Astronomy University of Maryland College Park MD USA; ^6^ Department of Geophysics Graduate School of Science Kyoto University Kyoto Japan

**Keywords:** Mars, magnetic reconnection, magnetosphere, crustal anomalies

## Abstract

The Martian crustal magnetic anomalies present a varied, asymmetric obstacle to the imposing draped interplanetary magnetic field (IMF) and solar wind plasma. Magnetic reconnection, a ubiquitous plasma phenomenon responsible for transferring energy and changing magnetic field topology, has been observed throughout the Martian magnetosphere. More specifically, reconnection can occur as a result of the interaction between crustal fields and the IMF, however, the global implications and changes to the overall magnetospheric structure of Mars have yet to be fully understood. Here, we present an analysis to determine these global implications by investigating external conditions that favor reconnection with the underlying crustal anomalies at Mars. To do so, we plot a map of the crustal anomalies' strength and orientation compiled from magnetic field data collected throughout the Mars Atmosphere and Volatile EvolutioN (MAVEN) mission. Then, we create “shear maps” which calculate and plot the angle of shear between the crustal fields and a chosen external field orientation. From there we define a “shear index” to quantify the susceptibility of a region to undergo reconnection based on a given overlaid, external field orientation and the resulting shear map for that region. We demonstrate that the shear analysis technique augments analysis of local reconnection events and suggests southward IMF conditions should favor dayside magnetic reconnection on a more global scale at Mars.

## Key Points

- This study assesses the conditions that favor high‐shear magnetic reconnection between an external magnetic field and the crustal magnetic anomalies at Mars
- Southward external fields are most likely to result in magnetic reconnection with the moderate‐to‐strong crustal fields globally
- This suggests large‐scale reconnection‐related phenomena such as the twisted magnetotail are organized by global preference for southward external field conditions

## 1 Introduction

Magnetic reconnection is a fundamental plasma process that governs the interaction and transfer of energy between plasma populations. On the dayside of an intrinsic planetary magnetosphere, magnetic reconnection between the interplanetary magnetic field (IMF) and the planetary field facilitates the transfer of energy between the Sun and the plasma environment of the planet, while changing magnetic topology and accelerating local plasma (e.g., Dungey, 1961; Slavin et al., 2010). The likelihood of reconnection to occur along a dayside magnetopause depends on the orientation of the magnetic fields adjacent to the boundary, among other factors. That is, reconnection is more likely to occur when the two magnetic field regimes are antiparallel, or highly sheared, to one another. For example, Earth possesses a global, intrinsic magnetic dipole field which points northward at the magnetic equator, and therefore exhibits a preference for southward IMF orientation for dayside magnetopause reconnection to occur (e.g., Crooker, 1979; Dungey, 1963). However, given the complexity of the crustal magnetic field environment around Mars, a more extensive analysis is required to determine the preferred conditions for reconnection to take place across the dayside magnetosphere.

The magnetosphere of Mars primarily differs from Earth's due to a lack of a global magnetic dynamo field. Instead, Mars possess crustal magnetic anomalies that are scattered across the surface of the planet (Acuña et al., 1999). These crustal fields protrude out into space creating many “minimagnetospheres” that comprise a dynamic and varied magnetic environment for the Mars‐solar wind interaction (Brain et al., 2003). Variations in the crustal field location due to diurnal and seasonal changes constantly alter the planetary obstacle to the solar wind. These nonuniform planetary fields, coupled with the dynamics of the system, lead to a much different interaction than what has been observed at intrinsic magnetospheres. In regions where the crustal magnetic fields are weak, the Martian conducting ionosphere acts as the primary obstacle to the solar wind flow, leading to the IMF draping around the planet (e.g., Luhmann et al., 2004; Ma et al., 2002). Despite the lack of a global, intrinsic magnetic field at Mars, observations of magnetic reconnection have been reported throughout the Martian magnetosphere (e.g., Brain et al., 2010; Cravens et al., 2020; Harada et al., 2018, 2020). Observations of flux ropes (Beharrell & Wild, 2012; Bowers et al., 2021; Brain et al., 2010; Briggs et al., 2011; Hara et al., 2014) and ion jets (Harada et al., 2020) at Mars are evidence of localized byproducts of magnetic reconnection between the crustal “minimagnetospheres” and a magnetic field external to Mars. Also, magnetic reconnection trends are thought to play a role in global phenomena such as the prevalence of open magnetic topology throughout the Martian magnetosphere (Brain et al., 2007, 2020; Dubinin et al., 2008; Lillis et al., 2011; Weber et al., 2017, 2020; Xu et al., 2014, 2019) and large‐scale closed magnetic loops that extend up to thousands of kilometers in altitude (Xu et al., 2017). These large‐scale phenomena suggest magnetic reconnection not only affects the plasma environment local to the reconnection region, but also may organize the cycling of open and closed magnetic fields on a more global scale.

Global dayside magnetic reconnection trends are also thought to play a role in the formation and structure of the Martian magnetotail. Recent studies using data from the Mars Atmosphere and Volatile EvolutioN (MAVEN) spacecraft (Jakosky et al., 2015) have revealed the magnetotail is twisted away from its expected location (DiBraccio et al., 2018, 2022) based on IMF draping expected of a purely induced magnetosphere, such as Venus (Luhmann et al., 2004). The terrestrial magnetotail also exhibits a twist based on the dawn‐dusk component of the IMF (e.g., Cowley, 1981; Kaymaz et al., 1994; Sibeck et al., 1985; Xiao et al., 2016). While the exact cause of the twisted terrestrial magnetotail is still debated, Cowley (1981) posited that dayside magnetic reconnection produces open field lines that exert a torque on the magnetotail, resulting in a twisted magnetotail configuration. Considering that dayside magnetic reconnection between the IMF and the terrestrial magnetic field is ultimately responsible for the twisted terrestrial magnetotail, DiBraccio et al. (2018, 2022) suggested that dayside magnetic reconnection between the crustal fields and draped IMF may be responsible for the twisted Martian magnetotail. The impact of dayside reconnection on the twisted magnetotail of Mars is an open‐ended question; however, similar investigations have been performed at Earth that provide tools to assess this possibility at Mars.

To analyze the draped IMF conditions favoring reconnection along the terrestrial magnetopause, Trattner et al. (2007a) developed the “maximum shear model.” This model calculates the magnetic shear angle between the draped IMF and the terrestrial field, both adjacent to the magnetopause boundary, to identify the location of reconnection X‐lines where magnetic reconnection occurs. These X‐line locations reveal regions that are likely to undergo “antiparallel reconnection,” where the magnetic fields internal and external to the magnetopause are highly sheared and pointed in opposite directions. Recent studies have demonstrated that X‐lines form along the magnetopause where the shear angle between the draped IMF and the magnetospheric magnetic fields are at a maximum and near 180° (e.g., Fuselier et al., 2017; Petrinec et al., 2016; Trattner et al., 2017). This approach has been used to assess the effect of varying IMF orientation on the occurrence of magnetopause reconnection and, therefore magnetic field circulation, at Earth. Additionally, the “maximum shear model” has been applied to better understand the location of X‐lines during flux transfer events (FTEs) (Petrinec et al., 2020) as well as multiple X‐line reconnection events along the terrestrial magnetopause (Fuselier et al., 2022). A similar shear angle argument has been applied to predict IMF conditions that favor reconnection along the magnetopause of Mercury (Slavin et al., 2012), Jupiter (Desroche et al., 2012), Saturn (Fuselier et al., 2020), Uranus (Masters, 2014), and Neptune (Masters, 2015) and Ganymede (Kaweeyanun et al., 2020). The utility of this analysis at Earth and other planetary objects has proven that the maximum shear model is a useful tool to assess conditions and predict locations of magnetic reconnection along a magnetopause. Although Mars presents a dynamic magnetic obstacle to the impinging IMF, a maximum shear model will help to better understand the conditions that drive the occurrence of reconnection as part of the Mars‐solar wind interaction. To achieve this, we must develop a tool that considers the complex crustal field geometry at Mars, including their location and strength as well as variations in the magnetopause altitude due to the nonuniformity of the magnetic environment. This maximum shear model for Mars will be the first of its kind to explore a global understanding of conditions that favor magnetic reconnection, which directly impact the structure and dynamics of the Martian magnetosphere.

Here, we present a maximum shear analysis of the Martian magnetosphere to assess which upstream IMF conditions favor the onset of magnetic reconnection with crustal fields on the dayside of Mars. We produce magnetic shear maps that highlight high‐shear regions between the Mars crustal fields and an external field, which are predicted to be more susceptible to antiparallel reconnection. We demonstrate the validity of this analysis by applying it to a previously reported reconnection event observed using in situ MAVEN data. We then apply the shear analysis technique more globally to determine the external field conditions that favor the onset of magnetic reconnection throughout the global magnetosphere of Mars. Our results provide a framework for understanding global reconnection trends at Mars and provide insight into outstanding questions regarding nightside magnetospheric activity, namely how IMF orientation affects the twisting of the magnetotail.

## 2 Methodology

### 2.1 MAVEN Data

We investigate external field‐crustal field reconnection through an analysis of data provided by the MAVEN Magnetometer (MAG) instrument (Connerney et al., 2015), which measures vector magnetic fields at a maximum sampling rate of 32 vectors/s. MAVEN's orbit precesses across a variety of local times (i.e., the time of a location with respect to the overhead position of the Sun), altitudes, and longitudes to provide a global coverage of the Martian space environment. This study generates crustal field maps utilized in the shear analysis by compiling MAG measurements collected on the nightside (solar zenith angle (SZA) > 90°), low‐mid altitude (150–800 km) passes of the Martian magnetosphere. Our analysis considers the magnetic field data in two coordinates systems: the spherical coordinate system [$\hat{R}$, $\hat{E}$, $\hat{N}$] in which $\hat{R}$ points radially outward from Mars, $\hat{E}$ points to the east, and $\hat{N}$ points to the north, and the Mars Solar Orbital (MSO) coordinate system [${\hat{X}}_{MSO}$, ${\hat{Y}}_{MSO}$, ${\hat{Z}}_{MSO}$], in which ${\hat{X}}_{MSO}$ points from the center of Mars toward the Sun, ${\hat{Z}}_{MSO}$ points toward geographic north, and the ${\hat{Y}}_{MSO}$ points along the ecliptic.

MAVEN plasma data are also analyzed to complement MAG observations and assess conditions for magnetic reconnection at Mars. Superthermal (>1 eV) electron energy distributions available from the Solar Wind Electron Analyzer (SWEA; Mitchell et al., 2016) are used to estimate the source of the electrons measured by the spacecraft (Xu et al., 2017). SWEA measures electron fluxes at a 4 s cadence, and we use these fluxes to parameterize the pitch angle distribution (PAD) of the electrons as demonstrated by Weber et al. (2017). We also compute ion velocity moments via 4 s resolution measurements taken by the SupraThermal and Thermal Ion Composition (STATIC) instrument (McFadden et al., 2015) onboard MAVEN which operates over an ion energy range of 0.1 eV–30 keV.

### 2.2 Martian Shear Analysis

The maximum shear model presented for the terrestrial case in Trattner et al. (2007a) calculates and plots the shear angle along the Earth's magnetopause between the Earth's intrinsic field and a draped IMF orientation to estimate the location of the X‐line formed via reconnection. This technique has been validated via multipoint in situ observations of reconnection events from the Cluster spacecraft (Fuselier et al., 2011) and the more recent Magnetospheric Multiscale Mission (e.g., Fuselier et al., 2017; Petrinec et al., 2016). We perform a similar analysis at Mars by first creating a data‐derived map of the crustal magnetic fields based on low‐midaltitude MAVEN MAG measurements of crustal anomalies on the nightside of the planet and then imposing an external field to calculate the shear angle between the two.

**Figure 1:**
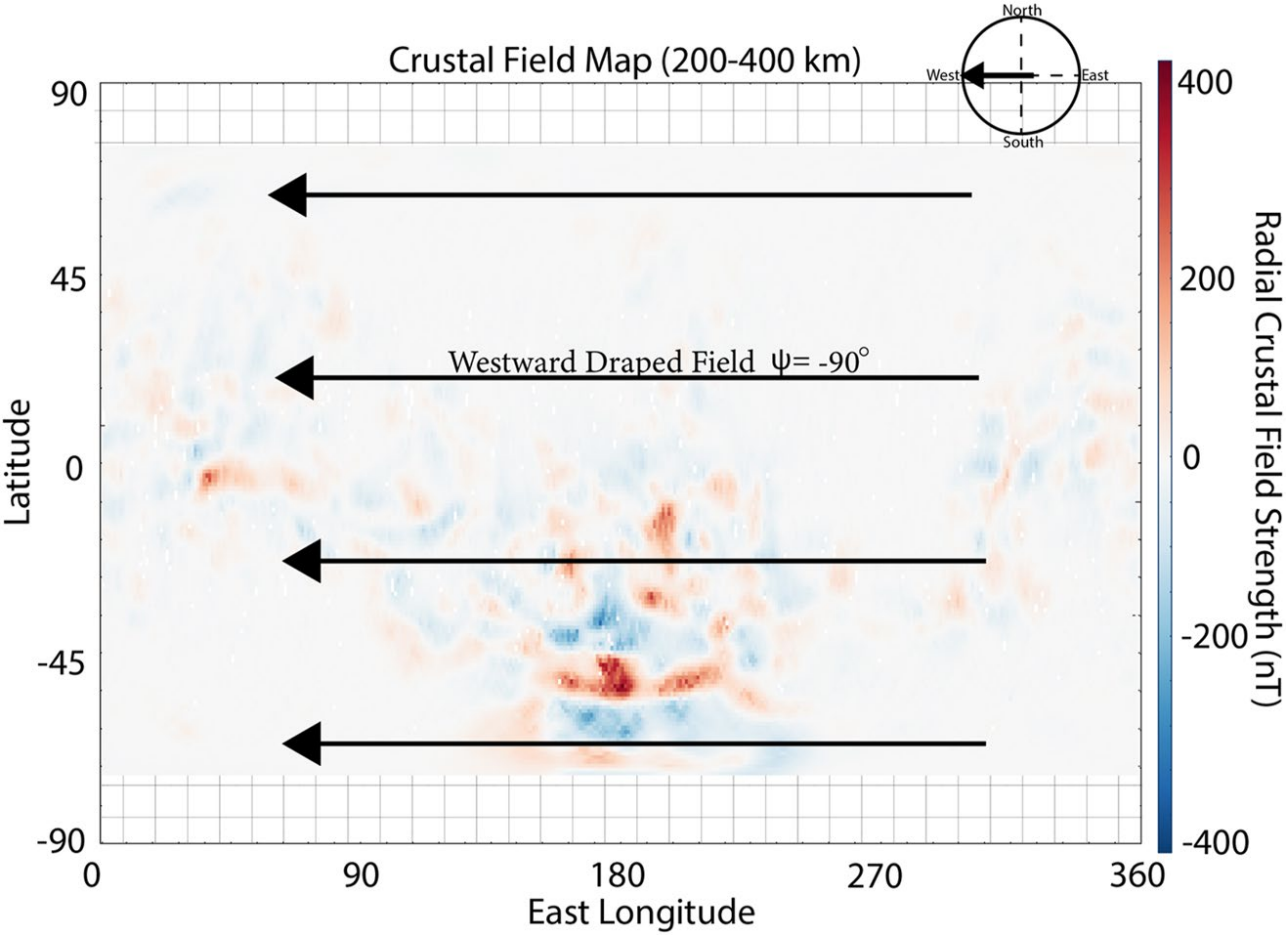
Map of crustal magnetic fields at Mars generated from Mars Atmosphere and Volatile EvolutioN (MAVEN) passes at altitudes ranging from 200 to 400 km colored by the radial magnetic field strength. The large, black arrows represent the direction of an example external field that is used to generate a shear map (westward in this case).

**Figure 2:**
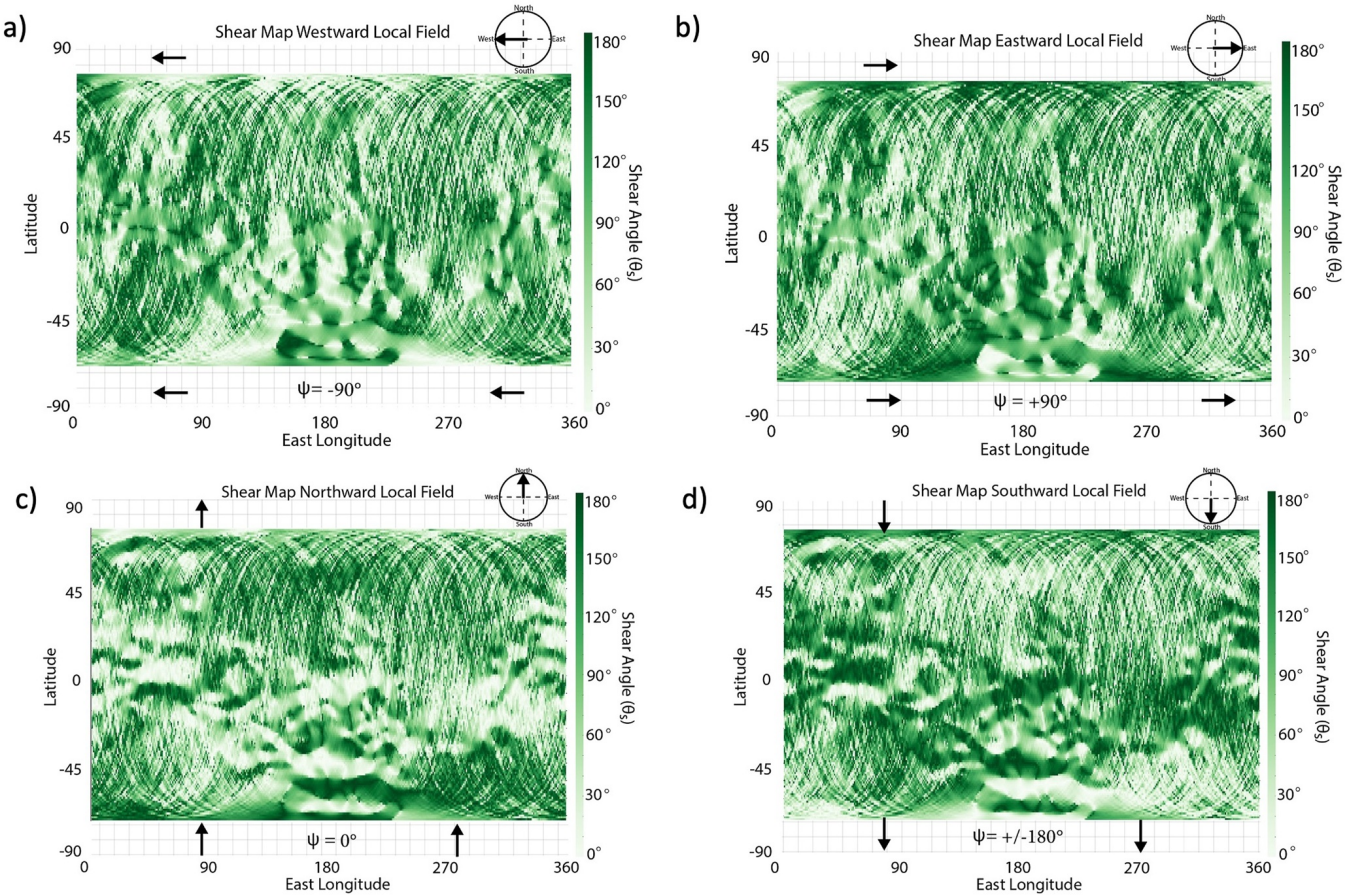
Shear maps of full data‐derived crustal field map for (a) westward (ψ = −90°), (b) eastward (ψ = +90°), (c) southward (ψ = ±180°), and (d) northward (ψ = 0°) external magnetic field orientation. The maps represent the crustal magnetic anomalies colored by the shear angle ($\theta_{s}$) calculated for the given external field direction, (c).

The formation of this Martian data‐derived crustal field map is described in detail in Weber (2020) and has been updated for this study to include the most recent MAVEN measurements, spanning 2014–2021. This data set is averaged into 1° longitude by 1° latitude bins. The Martian magnetopause varies in altitude due to the nonuniform strength of the crustal anomalies (Brain et al., 2005), and so the crustal field map has been separated into three altitude ranges: 200–400, 400–600, and 600–800 km. Due to MAVEN's orbital inclination, the crustal map only extends from 70°S to 70°N latitude. Only observations taken when MAVEN was on the nightside (SZA > 90°) of Mars were included to reduce the perturbations of crustal fields by external interactions due to changes in solar wind dynamic pressure (de Oliveira et al., 2021). Following the generation of the crustal field map, we overlay an external magnetic field across the entire map of the crustal anomalies (Figure 1). This external field orientation can be changed to test various conditions at Mars. The orientation of the external field is given by the local magnetic field clock angle ψ, where $\psi =\arctan\left(\frac{B_{E}}{B_{N}}\right)$ and $B_{E}$, $B_{N}$ are the eastward and northward components of the external magnetic field, respectively. The value of ψ spans from −180° to 180°, where ψ = ±180°, −90°, 0°, 90° corresponds to southward‐directed, westward‐directed, northward‐directed, and eastward‐directed external magnetic fields. Then, the shear map is generated by calculating the shear angle ($\theta_{s})$ between the underlying transverse component of the crustal anomalies and the overlaid, external magnetic field. Specifically, $\theta_{s}$ is calculated within each bin by taking the inverse cosine of the dot product of the external field with the underlying normalized component of the crustal field, i.e., transverse to the planet. Since we are performing a 2D analysis, we do not consider the impact of the radial component of the external field or crustal anomalies when calculating $\theta_{s}$. We note that the map of the underlying crustal anomalies may change for different altitude ranges but remains constant across shear maps within the same altitude range. This process determines the shear angle between the Martian crustal fields, at a selected altitude range from the three listed above, and the impinging external magnetic field for a given orientation (Figure 2). For example, the “shear maps” in Figure 2 are calculated by imposing a westward‐directed (Figure 2a), eastward‐directed (Figure 2b), northward‐directed (Figure 2c), and southward‐directed (Figure 2d) external magnetic field on top of the crustal field map generated from the 200 to 400 km altitude data.

The next step is to quantify the results of these shear maps by calculating a “shear index,” S, that determines the susceptibility of a given region to undergo reconnection based on the $\theta_{s}$ values within each bin in this region. Because we are aiming to identify regions that are likely to experience high‐shear, antiparallel magnetic reconnection, we consider bins with 150° < $\theta_{s}$ < 180° as high‐shear reconnection regions on the shear maps, which is the same definition of “high‐shear” described for the terrestrial maximum shear model (Trattner et al., 2007b, 2012). For a shear map generated with a given, S is defined within a region R as:

$$S=\frac{n_{i}}{N}$$

**Figure 3:**
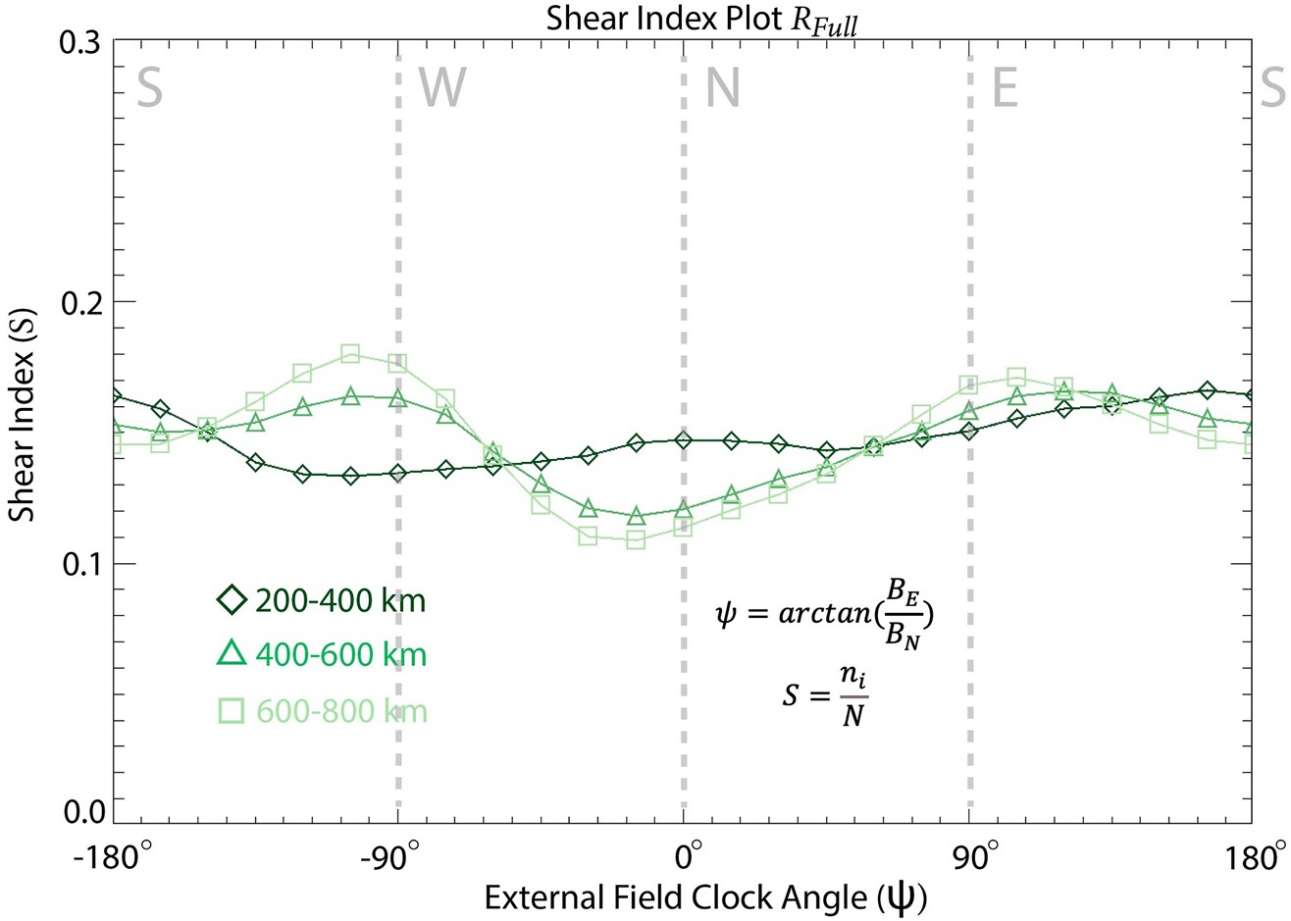
The magnetic shear index (*S*) within *R*_full_ across a variety of local draping clock angles, from crustal magnetic field data taken from three different altitude ranges: 200–400 km (diamonds), 400–600 km (triangles), and 600–800 km (squares).

Here, $n_{i}$ is the number of bins in which 150° ${<\theta }_{s}<$ 180° and N is the total number of bins. Thus, S represents a normalized value determined from the relative number of bins within a region R in which $\theta_{s}$ is large. S ranges from 0 to 1, with 0 indicating there are no high‐shear bins within R, and 1 indicating every bin within R is within the high‐shear range (150° ${<\theta }_{s}<$ 180°). Figure 3 is a “shear index plot,” which plots S versus ψ, for the three altitude ranges across the region $R_{full}$, where $R_{full}$ covers the entire crustal field map data set from 0 to 360° longitude and 70°S–70°N latitude (50,400 total bins). In short, the shear maps shown in Figures 2a–2d visualize magnetic shear variations across the crustal field map data set for a single ψ, whereas the shear index plot shown in Figure 3 highlights trends in S across all values of ψ within the region $R_{full}$, to determine which ψ direction is likely to generate a larger occurrence of reconnection. This shear index plot will be discussed in more detail in Section 3.2.

In comparison to the Trattner et al. (2007a) maximum shear model for the terrestrial case with the analysis for the Martian crustal fields presented here, Trattner et al. (2007a) used two models to estimate the orientation of the magnetic field lines in the terrestrial environment: (a) the Tsyganenko and Stern (1996) model, which estimates the orientation of Earth's magnetospheric field along the magnetopause and (b) the Cooling et al. (2001) model, which estimates the orientation of the draped IMF. In contrast, our study creates and utilizes a data‐derived map to determine the strength and orientation of the crustal fields, and then overlays a single external magnetic field orientation across the map to calculate the local shear angles. We note that unlike the terrestrial maximum shear model, draping effects are not taken into consideration for our study because the draping interaction at Mars is not straightforward due to the nonuniform crustal magnetic fields. Also, the crustal magnetic fields may interact with external fields that are not the draped IMF, including open magnetic fields. As a result, our analysis is two‐dimensional and provides an understanding of the local interaction between a given crustal field region with an overlaid, external field orientation, so we can elucidate which orientations are more susceptible for high‐shear magnetic reconnection to occur.

The terrestrial analysis is comprised of two models and must be performed in different coordinate systems. The modeled IMF draping around Earth's magnetopause ensures that the Geocentric Solar Magnetospheric (GSM) coordinate system (${\hat{X}}_{GSM}$ points sunward, ${\hat{Z}}_{GSM}$, points along the dipole axis, and ${\hat{Y}}_{GSM}$ completes the right‐hand coordinate system) is the best option for the terrestrial maximum shear model. However, no sophisticated model of the draped IMF around Mars across all local times and possible crustal field configurations currently exists. As a result, our shear analysis focuses on the local interaction between crustal fields and external fields and is therefore performed in the spherical coordinate system defined in Section 2.1. Future studies are needed to incorporate a model of the complex draping of the IMF around Mars to better understand the upstream IMF conditions in the MSO frame that would favor reconnection with the crustal anomalies.

High shear between the crustal fields and the external field is a necessary but not sufficient condition for magnetic reconnection at Mars. Dayside reconnection along the terrestrial magnetopause occurs tens of thousands of kilometers away from Earth where the plasma is collisionless. On the other hand, an external field and the Martian crustal fields may interact within the Martian ionosphere where collisional effects may take place. In this regime, ion‐neutral collisions affect the onset of reconnection by limiting the flow into and out of the diffusion region and by decreasing the magnetic Reynold's number. Cravens et al. (2020) explored the implication of ion‐neutral collisions on the onset magnetic reconnection within the Martian ionosphere by considering a current sheet of the typical length scale of a crustal anomaly (L≈ 300 km) and the average dayside Martian atmospheric density profile provided by Cravens et al. (2017). They concluded that collisional effects should slow the dayside rate of reconnection at altitudes below ∼300 km, above which the effects of ion‐neutral collisions on reconnection rates are negligible. Moreover, Harada et al. (2020) surveyed low‐altitude current sheets in the Martian ionosphere to identify reconnection signatures, including accelerated ion jets and Hall magnetic fields. Ion jets accelerated away from the reconnection X‐line are a primary biproduct of magnetic reconnection and are a clear example of the transfer from magnetic energy to kinetic energy of the surrounding particles (Paschmann et al., 1979, 2013). Harada et al. (2020) identified ion jets associated with magnetic reconnection within current sheets were identified down to 200 km in altitude. Results from both Cravens et al. (2020) and Harada et al. (2020) suggest the plasma conditions for magnetic reconnection are frequently met within the altitude ranges analyzed in Figure 3. As a result, in this study we assess the shear angle condition responsible for the onset of magnetic reconnection on the dayside of Mars.

## 3 Results

### 3.1 Local Crustal Field Reconnection

**Figure 4:**
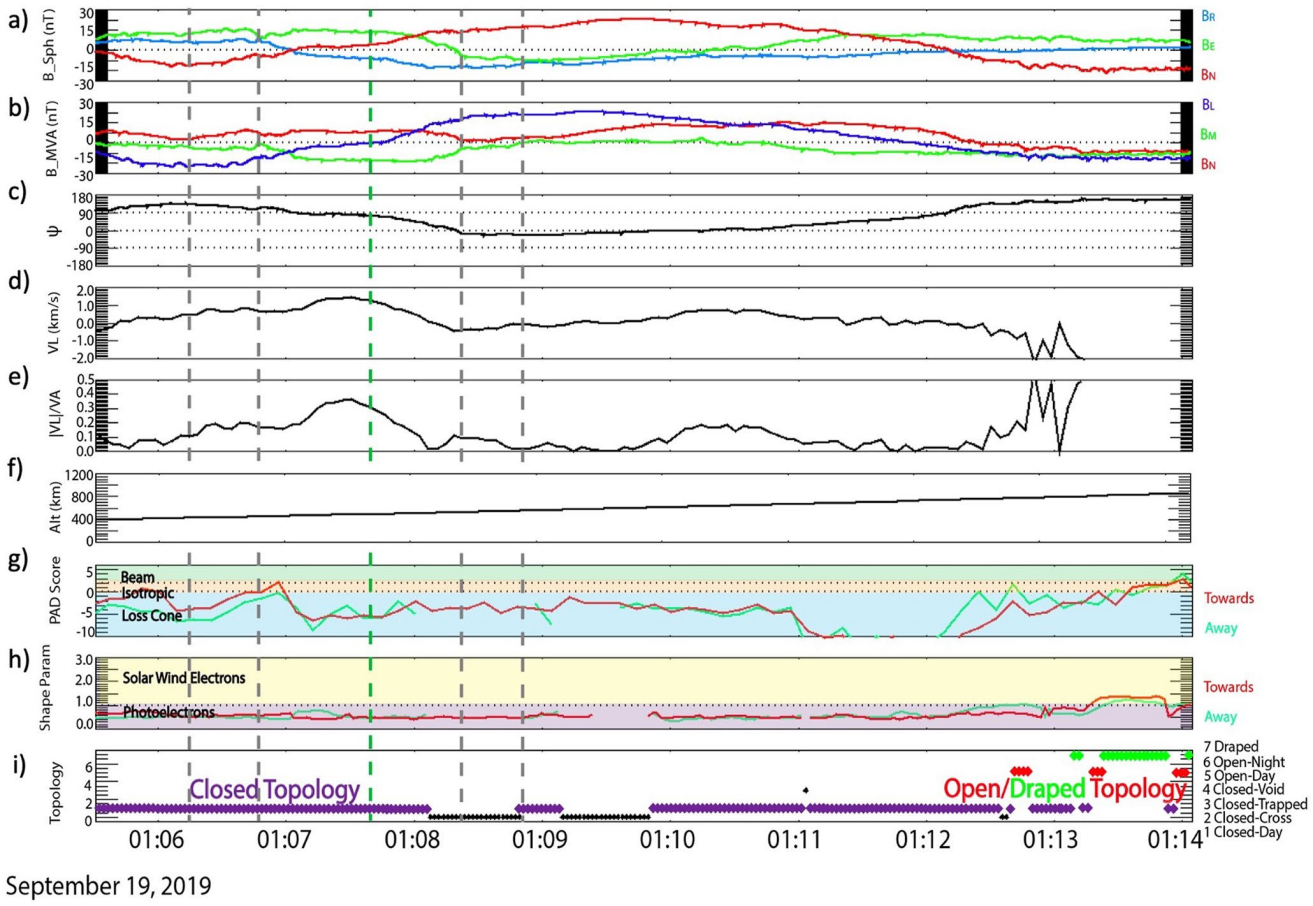
Mars Atmosphere and Volatile EvolutioN (MAVEN) observations on 19 September 2019 from 01:05:40 to 01:14:00 UT of the magnetic field in Mars Solar Orbital (MSO) frame (nT) (a), magnetic field in spherical frame (nT) (b), magnetic field in minimum variance analysis (MVA) frame (nT) (c), field local clock angle ψ (d), L component of the averaged ion velocity (km/s) (e), ratio of averaged ion velocity magnitude in *L* direction to local Alfvén velocity (f), altitude (g), pitch angle distribution (PAD) score (h), shape parameter (i), and magnetic topology score (j).

**Figure 5:**
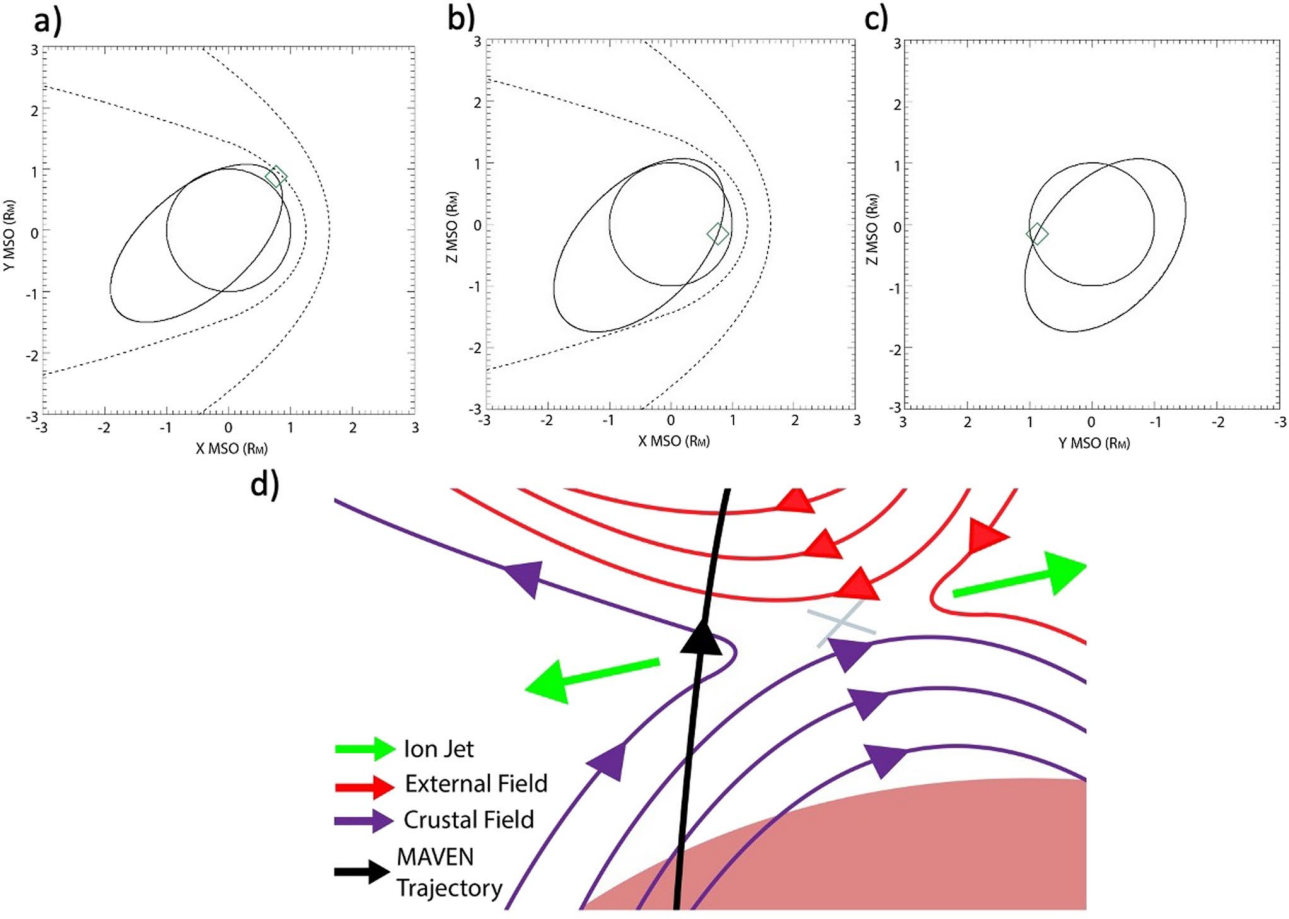
Mars Atmosphere and Volatile EvolutioN (MAVEN) orbit as viewed from the *Z*_MSO_ plane (a), *Y*_MSO_ plane (b), and *X*_MSO_ (c) plane. The green diamond in panels (a–c) represent the location of the current sheet. The dashed lines in panels (a–c) show the nominal locations of the bowshock and magnetic pileup boundary from Trotignon et al. (2006). Schematic of reconnection region and ion jet outflow with colored arrows representing the ion jet (green), crustal magnetic field orientation (purple), external field orientation (red), and the trajectory of the spacecraft (black) (d).

We apply the shear analysis described in Section 2 to a local region surrounding a magnetic reconnection event observed as part of a statistical analysis performed by Harada et al. (2020). Figure 4 shows MAVEN particle and fields data from a current sheet crossing included in the Harada et al. (2020) study from 19 September 2019, when the spacecraft was positioned ∼1,600 local time and near the equatorial crustal magnetic fields (Figures 5a–5c). The current sheet crossing is denoted by a reversal in $B_{N}$ at 01:07:45 UT when the field component changed from −30 nT to +30 nT (Figure 4a). This reversal can also be observed in the minimum variance analysis (MVA; Sonnerup & Scheible, 1998) coordinate system (Figure 4b) in which the maximum variance direction ($\hat{L}$) points along the antiparallel field direction that defines the two lobes of the current sheet. Figure 4c shows the ∼180° rotation in ψ defined above. We calculate the ion mass flow by averaging the velocities of the H^+^, O^+^, and O_2_^+^ ions measured by STATIC and weighted by their mass densities. The enhancement in this averaged ion velocity along the $\hat{L}$ direction ($V_{L}$) near the current sheet structure is a characteristic sign of an ion jet accelerated away from a reconnection X‐line (Figure 4d; Harada et al., 2020). Figure 4e plots the ratio between | $V_{L}$ | and the average Alfvén velocity within the inflow regions of the current sheet denoted by the gray lines, which shows the ion jet reaches speeds up to around 40% of the Alfvén velocity.

By determining the magnetic topology of the fields within and surrounding the current sheet crossing, it is possible to confirm that reconnection occurred between an underlying crustal anomaly and an external magnetic field, rather than some other combination of magnetic field line topologies that might result in reconnection (i.e., induced magnetopause reconnection; Wang et al., 2021). The pitch angle distribution score (PAD score; Figure 4g) and the shape parameter (Figure 4h) are quantities that aim to parameterize the distribution (loss cone, isotropic or beam‐like) and origins (solar wind electrons or photoelectrons) of the electrons measured by SWEA. These parameters combine to estimate magnetic topology (Weber et al., 2017; Xu et al., 2017, 2019; Figure 4i), which distinguishes among closed (both ends connected to the planet), open (one end connected to the planet and the other connected to the IMF) and draped (both ends connected to the IMF) field lines. As the spacecraft increased in altitude (Figure 4f), MAVEN exited a plasma regime dominated by photoelectrons, with an isotropic PAD and closed‐dayside magnetic topology (∼01:05:30–01:12:40) and entered a plasma regime populated by a mixture of solar wind and photoelectrons, with a beam‐like distribution and open‐dayside magnetic topology (01:12:40–01:14:00). This transition was detected ∼6 min after the magnetic field reversal and accelerated ion jet. This change in field topology with the reconnection signatures identified by Harada et al. (2020) suggests that MAVEN observed a reconnection event between a closed crustal anomaly and an external magnetic field. This idea is also supported by the orbital position of the spacecraft, where MAVEN was near the nominal location of the induced magnetopause between the magnetosheath and the ionosphere (Figures 5a–5c) on the dayside. The schematic shown in Figure 5d illustrates the likely scenario of this reconnection event, including the direction of the ion jet and the sampling of different magnetic topologies along the spacecraft's trajectory. We note that reconnection accelerates electrons, and may disrupt their energy distributions or PADs, leading to an error in the topology score. However, the details of this process would likely lead to a topology score of “draped” when the field line was actually open. Both open and draped magnetic field lines are classified as external to the underlying crustal anomalies, so distinguishing between the two is unimportant for this shear analysis.

**Figure 6:**
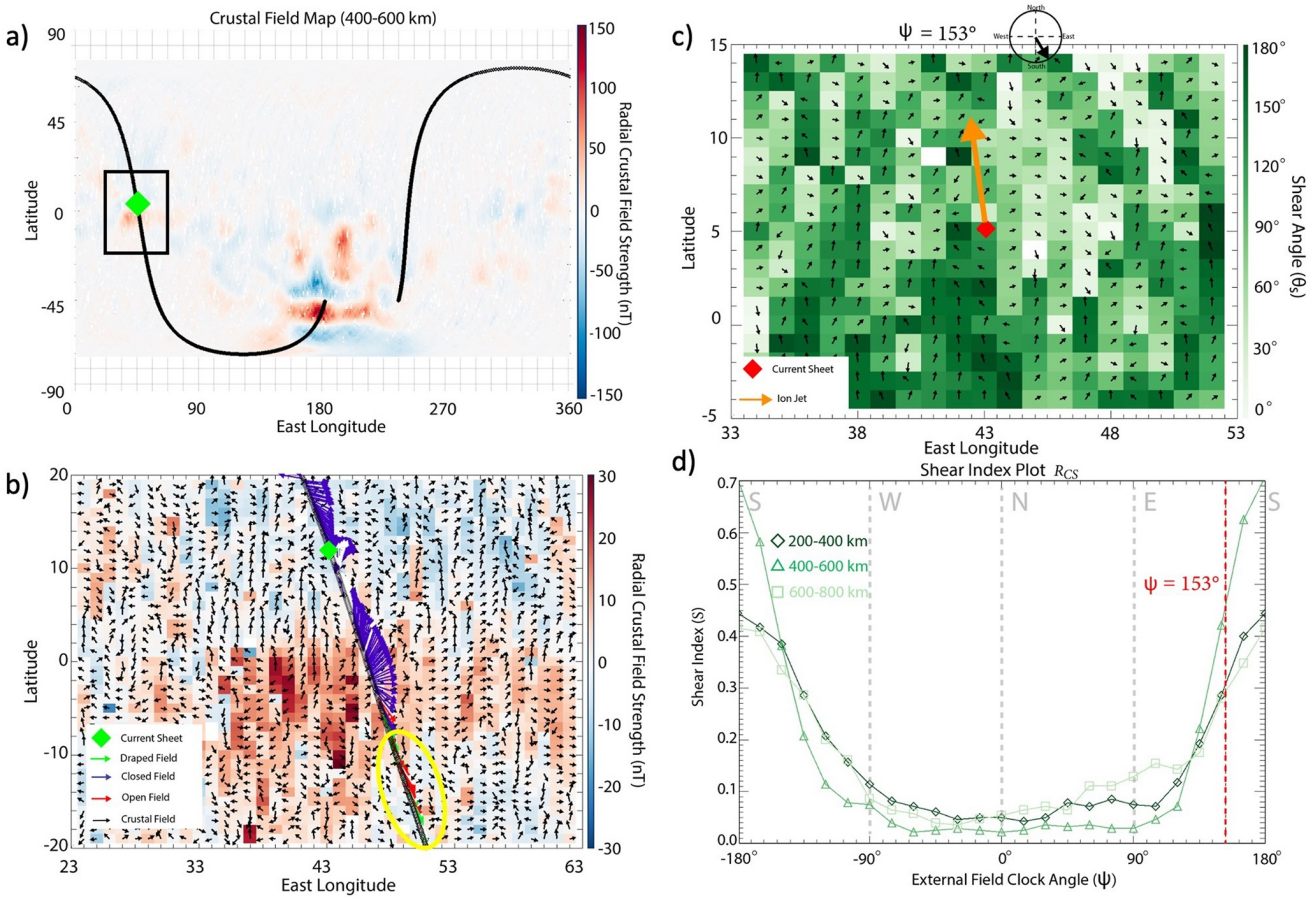
Shear analysis of the region surrounding the current sheet presented in Figure 4: Mars Atmosphere and Volatile EvolutioN (MAVEN) orbital trajectory of projected onto data‐derived map of crustal magnetic fields at 400–600 km altitude (a), projection of MAVEN trajectory through zoomed‐in crustal magnetic field region with colored arrows representing the topology of the magnetic field (closed‐purple, open‐red, unknown‐white, draped‐green), a green diamond representing the current sheet and yellow oval representing the open/draped magnetic field measurements (b). Shear map of $R_{CS}$ for an external draped field orientation of $\psi_{cs}$ = 153° and red diamond indicating current sheet location and orange arrow representing the projected ion jet direction (c). Shear index plot for this region with $\psi_{cs}$ indicated by a red dashed line (d).

By establishing that this reconnection event likely occurred between closed crustal fields and an external field, we are able to test the validity of the shear analysis. Figure 6a shows MAVEN's orbital trajectory over the data‐derived crustal field map, within the altitude range of 400–600 km. The current sheet crossing (green diamond) took place near the equator. Figure 6b shows a zoomed‐in subset of Figure 6a: a 40° by 40° box centered on the equator near the location of the current sheet. The arrows plotted along the trajectory align with the measured magnetic field direction in the spherical coordinate system and are colored by the magnetic topology score. We determine the external field orientation of this current sheet ($\psi_{cs}$) by averaging the orientation of the open/draped field lines detected by MAVEN around this region along its orbit ($\psi_{cs}$ = 153°) and produce a shear map of a region $R_{CS}$, defined as the 20° by 20° box centered on the current sheet, for the overlaid external field orientation $\psi_{cs}$ condition (Figure 6c). This shear map illustrates the large range in $\theta_{s}$ within this region, with the highest shear occurring when the crustal anomalies point northward. We also project the direction of the ion jet onto the shear map shown in orange. Note that the ion jet points away from the reconnection X‐line and in this case, points north and slightly west which suggests the X‐line lies somewhere just southeast of the current sheet detection location. This is reflected in the shear map with the highest shear bins located southward of the current sheet detection location.

We perform the shear index analysis within $R_{CS}$ and plot the results in Figure 6d. The peak values of S within this region are at ψ∼±180° across the 200–400, 400–600, and 600–800 km altitude ranges. These results are in good agreement with the measured value of $\psi_{cs}$ = 153°, particularly for the midaltitude range in which this event is observed (∼500 km, Figure 4f). This small‐scale study shows the shear analysis predictions provide context for the interaction of an external field and the crustal anomalies to augment single‐point measurements taken by the spacecraft. Furthermore, this study suggests the shear analysis technique can provide insights into the location and preferred external field orientation for magnetic reconnection with the crustal anomalies throughout the magnetosphere.

### 3.2 Global Magnetospheric Reconnection

We apply this shear analysis methodology more globally to explore the external field conditions that favor the onset of magnetic reconnection throughout the Martian magnetosphere. Figures 2 and 3 demonstrate the shear analysis results for the entire data‐derived crustal field map, $R_{full}$. The shear index results in Figure 3 vary across the three altitude ranges: For the two higher altitude ranges, S peaks (S≈ 0.17) at ψ values close to −90° (westward) and +90° (eastward), while S values for ψ close to 0° (northward) reach ∼70% (S≈0.12) of eastward and westward S values. We also see that S values for northward fields reach only ∼80% of those for southward (ψ = ±180°) fields (S≈ 0.15) for the higher two altitude ranges. At the lowest altitude range S does not show as much variation with ψ, but does exhibit a small peak (S≈ 0.16) for southward external fields. These results provide a global perspective of the external conditions under which high shear, antiparallel magnetic reconnection is likely to occur.

**Figure 7:**
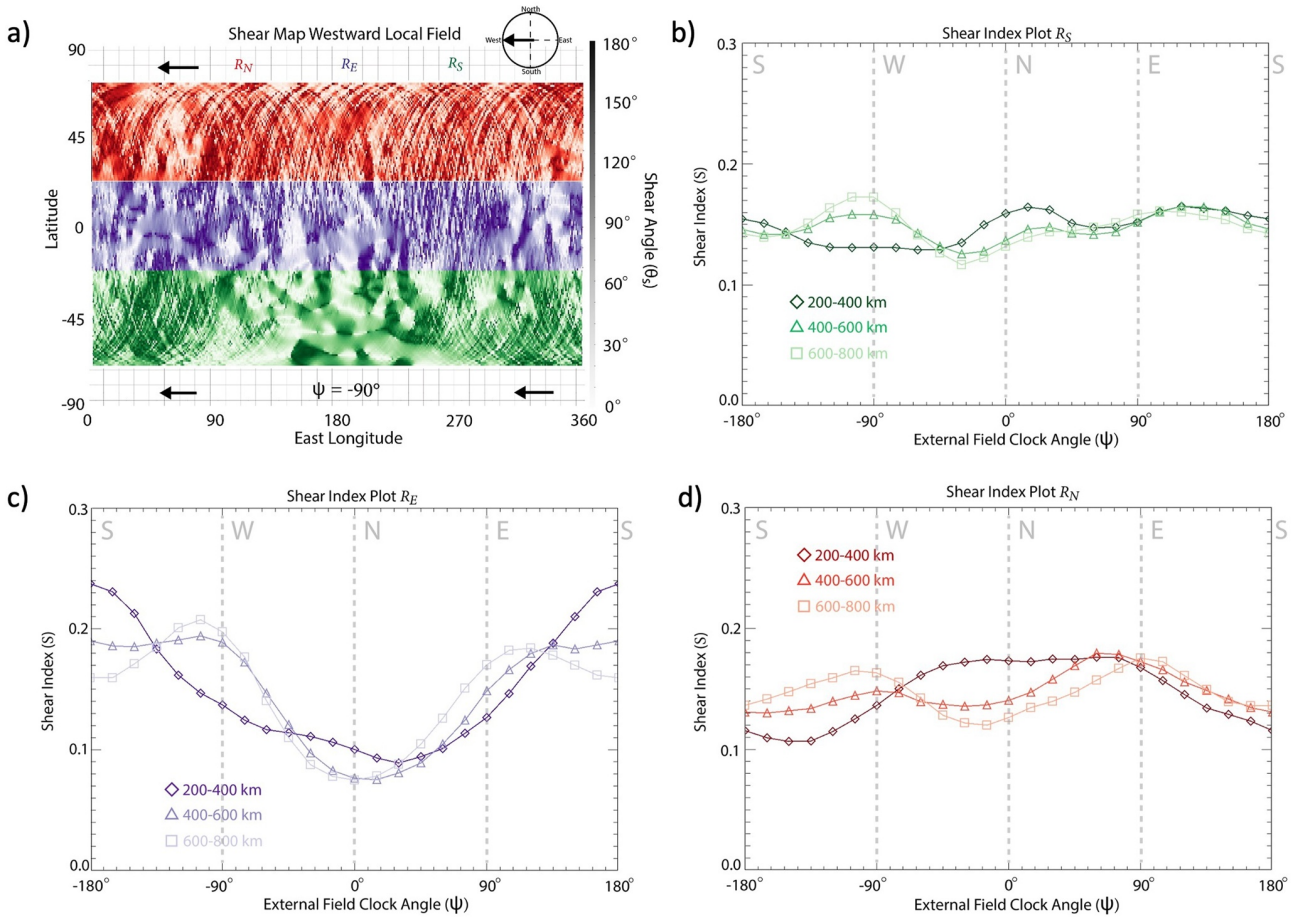
Shear analysis plots for $R_{N}$, $R_{E}$, $R_{S}$ for the data‐derived crustal field map. (a) Shear map of crustal magnetic fields for a westward draped field orientation measured from 400 to 600 km altitude split into three latitude ranges ($R_{N}$: +20° to +70°, $R_{E}$: −20° to +20°, $R_{S}$: −70° to +70° with $R_{N}$, $R_{E}$, $R_{S}$ colored red, purple, and green, respectively). Shear index plots for $R_{N}$ (b), $R_{E}$ (c), $R_{S}$ from crustal magnetic field data measured within three different altitude ranges: 200–400 km (diamonds), 400–600 km (triangles), and 600–800 km (squares).

**Figure 8:**
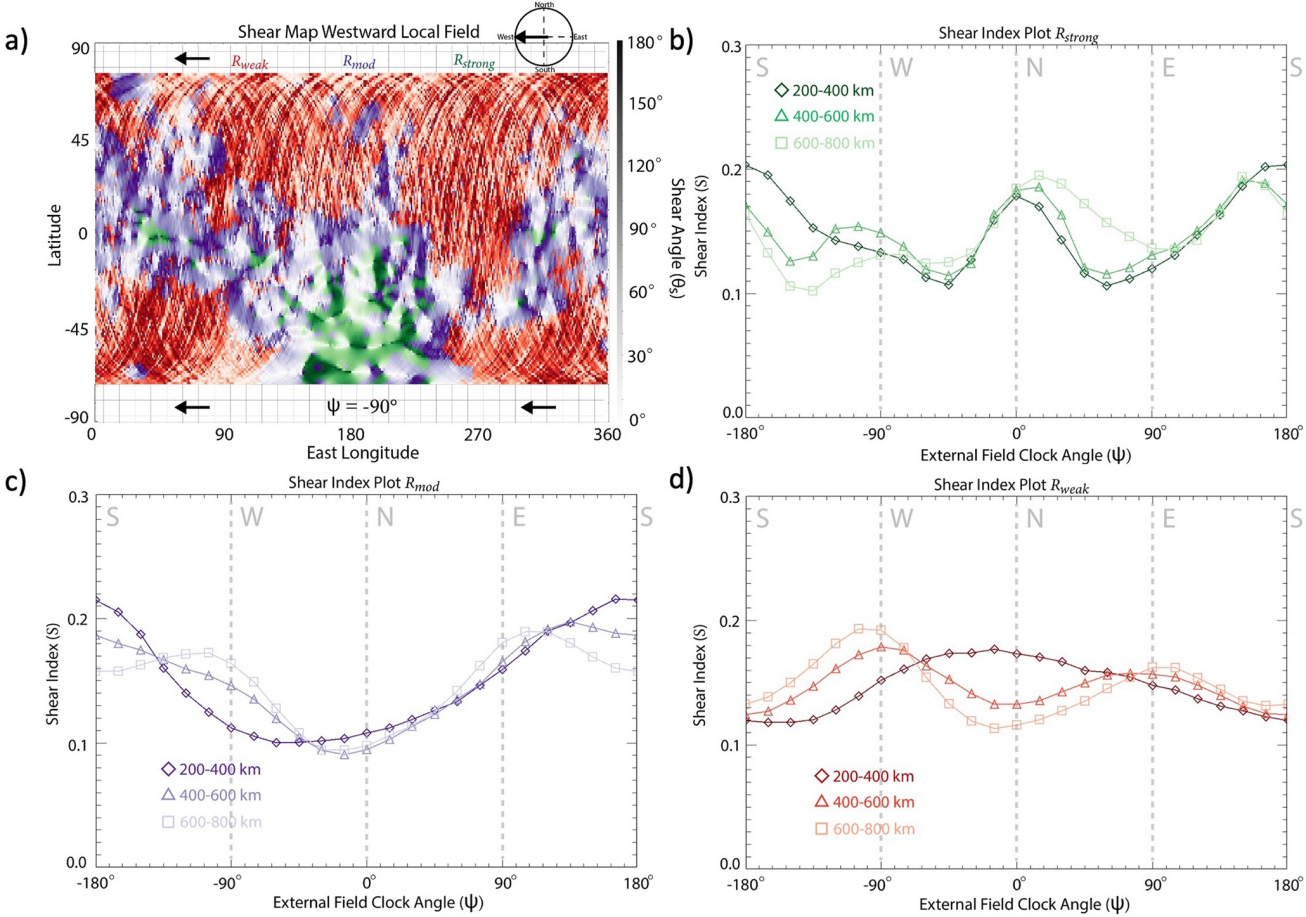
Shear analysis plots for $R_{weak}$ *,* $R_{med}$ *,* $R_{strong}$ for the data‐derived crustal field map. (a) Shear map of crustal magnetic fields for a westward draped field orientation measured from 200 to 400 km altitude split into three regions based on crustal magnetic field strength measured at altitudes <250 km ($R_{weak}$ *:* ${|B}_{cf}|$< 20 nT, $R_{med}$: 20 nT *<* ${|B}_{cf}|<$ 150 nT, $R_{strong}$:150 nT *<* ${|B}_{cf}|$) with $R_{weak}$ *,* $R_{\mathrm{mod}}$ *,* $R_{strong}$ colored red, purple and green respectively. Shear index plots for $R_{weak}$ (b), $R_{\mathrm{mod}}$ (c), and $R_{strong}$ (d) from crustal magnetic field data measured within three different altitude ranges: 200–400 km (diamonds), 400–600 km (triangles), and 600–800 km (squares).

It should be noted that while the shear index analysis assesses the relative orientations of the external field and the crustal anomalies, it does not consider any effects due variations in crustal field distribution or strength. To determine any dependencies on these parameters, we perform the shear analysis as a function of geographic location (Figure 7) and crustal field magnitude (Figure 8). Figure 7a shows the results of a shear map with an imposed westward external field with the map separated into three latitudinal regions: −70°S $\leq R_{S}\leq -$20°S (green region), 20°S $<R_{E}\leq$ 20°N (purple region), and 20°N $<R_{N}\leq$ 70°N (red region). The corresponding *S* plots for each latitudinal region over a range of ψ are displayed in Figures 7b–7d. Within $R_{S}$, S values do not show a clear dependence on ψ. This region includes the strongest crustal sources and, therefore, the largest variation in magnetic field sources compared to the other latitudinal regions (Figure 7b). When comparing *S* for the three regions, the S index plot for $R_{E}$ (Figure 7c) shows the most variability with altitude. Within $R_{E}$, a strong enhancement is observed in S for a southward external field (ψ=±180°) at low altitudes, maximizing at S∼ 0.25. The S values reach a local minimum for northward external fields (ψ=0°) across all altitudes, with S calculated to be ∼0.08 or ∼30% of that for southward draped fields. For $R_{N}$ (Figure 7d), 𝑆 values are larger for eastward/westward external magnetic fields compared to northward/southward external magnetic fields for high altitudes, similar to the shear index plot for the global magnetosphere (Figure 3). However, at the lowest altitude range, S peaks at S≈ 0.17 for northward external fields, with S for southward external fields reaching only 0.10 or ∼58% of that for a northward external field.

To explore the effects of crustal field strength on magnetic reconnection occurrence, we divide the magnetosphere into regions of crustal magnetic field amplitude (| $B_{cf}$ |) derived from the crustal field map data set measured at the lowest altitudes (<200 km): $R_{strong}$ (${150\;nT<\;|B}_{cf}|$), $R_{\mathrm{mod}}$ (${20\;nT<|B}_{cf}|<150\;nT$ ), $R_{weak}\;({|B}_{cf}|<20\;nT$), shown in Figure 8a. The corresponding shear index plots are shown in Figures 8b–8d. The *S* plot for $R_{strong}$, shown in Figure 8b is highly variable across the three altitude ranges. The $R_{strong}$ shear index plot shows S values that are larger for northward/southward external field lines (S≈ 0.2) compared to eastward/westward external field lines (S≈ 0.12). Figure 8c shows the shear index for region defined by the moderate strength crustal magnetic fields ($R_{\mathrm{mod}}$). At lower altitudes, strong peak in S is observed for southward fields (S≈ 0.22) with S for northward fields (S≈ 0.09) only reaching 40% of that for southward fields at all altitudes. The *S* plot for $R_{weak}$ (Figure 8d) reveals a bimodal trend at high altitudes, with a larger S measured for eastward/westward external fields compared to northward/southward external fields across all altitudes with a preference for westward external fields (S≈ 0.19) compared to eastward (S≈ 0.15). At the lowest altitude range, however, we see a peak in S for northward external fields (S≈ 0.17).

### 3.3 The Spherical Harmonic Model

**Figure 9:**
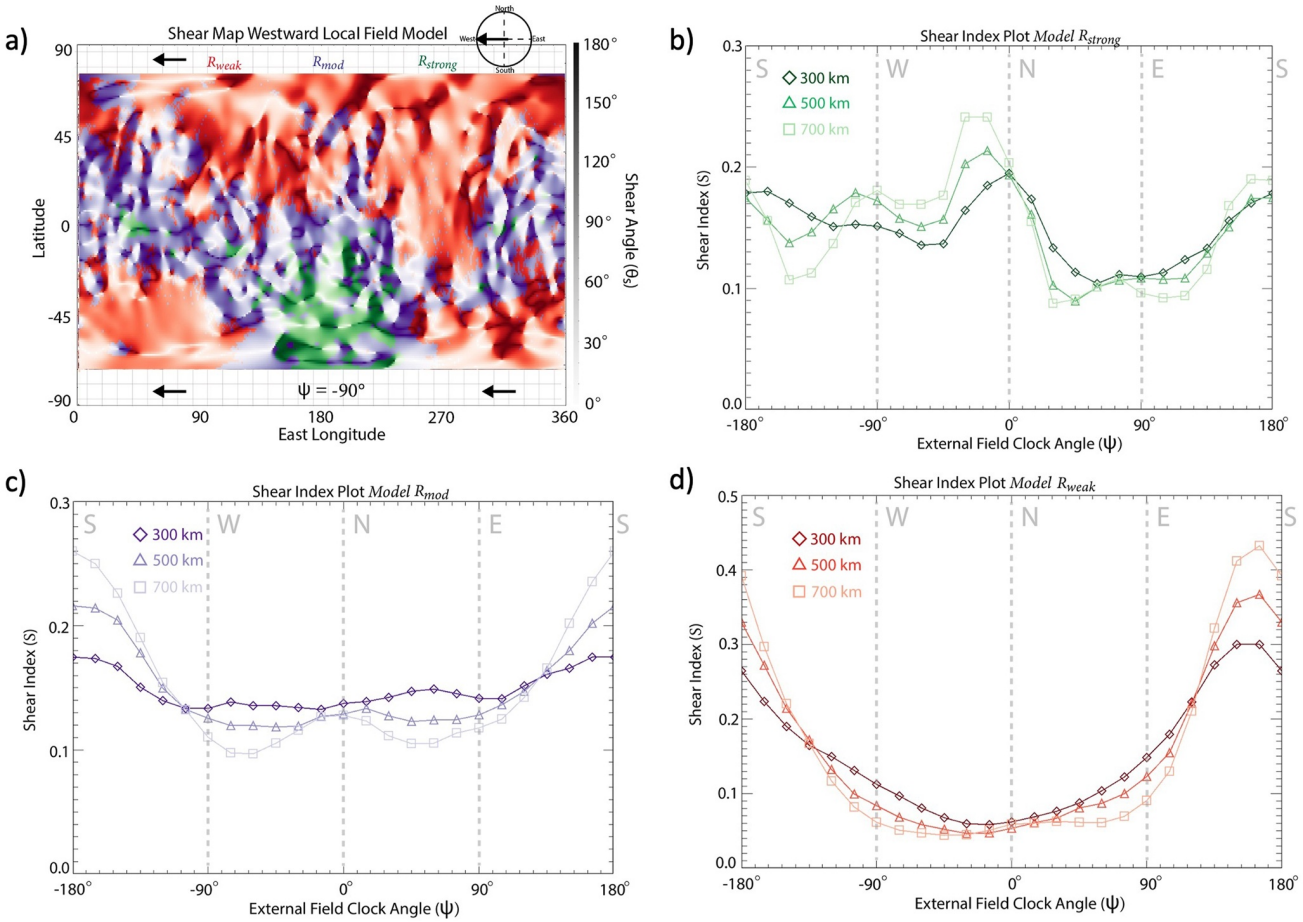
Shear analysis plots for $R_{weak}$, $R_{med}$, $R_{strong}$ for the crustal field spherical harmonic model described in Langlais et al. (2019). (a) Shear map of modeled crustal magnetic fields for a westward draped field orientation at 300 km altitude split into three regions based on crustal magnetic field strength measured at altitudes <250 km ($R_{weak}$: ${|B}_{cf}|$ < 20 nT, $R_{med}$: 20 nT< ${|B}_{cf}|<$ 150 nT, $R_{strong}$:150 nT <  ${|B}_{cf}|$) with $R_{weak}$, $R_{\mathrm{mod}}$, $R_{strong}$ colored red, purple, and green, respectively. Shear index plots for $R_{weak}$ (b), $R_{\mathrm{mod}}$ (c), and $R_{strong}$ (d) from crustal magnetic field data measured within three different altitude ranges: 300 km (diamonds), 500 km (triangles), and 700 km (squares).

We further explore our findings by implementing the shear analysis technique with the Langlais et al. (2019) spherical harmonic crustal field model (Langlais19) in place of the MAVEN data‐derived map. In this case, the methodology for producing the shear maps and S remains the same, but the crustal field strength and orientation is instead determined by Langlais19 rather than the data‐derived map. Figures 9a–9d shows the shear map and S plots for the $R_{strong}$, $R_{\mathrm{mod}}$, and $R_{weak}$ regions of crustal magnetism based on Langlais19. For $R_{strong}$, S peaks (S≈0.2–0.24) for northward/southward fields across all altitudes (Figure 9b), with eastward fields (S≈ 0.1) only reaching ∼40%–50% of northward/southward fields. For $R_{\mathrm{mod}}$, we see a peak in S for southward fields across all altitudes (Figure 9c). At the highest altitude range, S reaches values of 0.26. The shear index plot for the modeled crustal fields within $R_{weak}$ (Figure 9d) shows a major peak in S for southward draped fields (S≈ 0.45) across all altitudes, with S for northward fields (S≈ 0.05) reaching only 12% of that for southward fields.

## 4 Discussion

By developing a magnetic shear tool to investigate favorable conditions for magnetic reconnection between an external field and the Martian crustal fields occur, we can better understand how the IMF‐crustal field interaction drives overall magnetospheric structure and dynamics at Mars. The localized reconnection study detailed in Section 3.1 demonstrates how this Mars shear analysis augments single‐point measurements of magnetic reconnection by identifying crustal field regions where X‐lines are more likely to be located. A future application of the shear analysis on more localized scales includes analyzing the occurrence of other byproducts of magnetic reconnection such as magnetic flux ropes. The global application of the shear analysis in Section 3.2 serves to demonstrate where and under what conditions magnetic reconnection may occur across the dayside of the Martian magnetosphere. These results provide implications regarding the global magnetospheric interaction between the crustal anomalies of Mars and an external magnetic field to determine which conditions may drive periods of enhanced reconnection activity. Section 3.3 shows the S plots generated by utilizing the Langlais19 spherical harmonics crustal field to identify differences in trends between MAVEN observations and a commonly used crustal field modeling technique. Notable similarities and differences arise when comparing the *S* plot results of the data‐derived crustal field map versus the Langlais19 spherical harmonics map.

In comparison to the S plots generated utilizing the data‐derived crustal map (Figure 8), those generated utilizing Langlais19 are similar for $R_{strong}$ and $R_{\mathrm{mod}}$ (Figure 8, 9 and Figure 8, 9). The main difference between the two approaches is observed in regions of weak crustal fields. The data‐derived S plot peaks for northward external field orientations at low altitudes, and eastward/westward draped fields at higher altitudes for $R_{weak}$ (Figure 8d), whereas the Langlais19 S plot peaks majorly for southward fields (Figure 9d) across all altitudes. This discrepancy between the predictions of weak crustal magnetic field orientations from the model and those derived from MAVEN MAG measurements is likely due to a combination of three possibilities:

1. The lower order spherical harmonic terms in Langlais19 predict a more structured magnetic field orientation over the weak crustal anomalies than is actually present at Mars.
2. The regions of the weakest anomalies are heavily influenced by draped and induced magnetic fields, resulting from the lower magnetic pressure present in these regions. These draped and induced fields then affect the results based on the data‐derived maps.
3. Spacecraft data do not yet exist at low altitudes in these weak regions. MAVEN offers the lowest altitude magnetic field measurements of any Mars orbital mission to date and, therefore, the strength and direction of any weak crustal fields in this region may still be unexplored. This lack of data also affects the ability to model crustal fields in these weak regions.

Due to these considerations and the disagreement between the model and observations, we cannot make any strong conclusions regarding the external field conditions that favor reconnection with the weakest crustal anomalies within $R_{weak}$. Furthermore, because the weakest crustal anomalies are likely suppressed to such low altitudes, well within the Martian upper atmosphere, the moderate‐to‐strong anomalies play a larger role in global solar wind and magnetic reconnection trends at Mars. Further work is required to better understand the true orientation of the weakest crustal anomalies and their role, if any, in global trends of magnetic reconnection.

**Figure 10:**
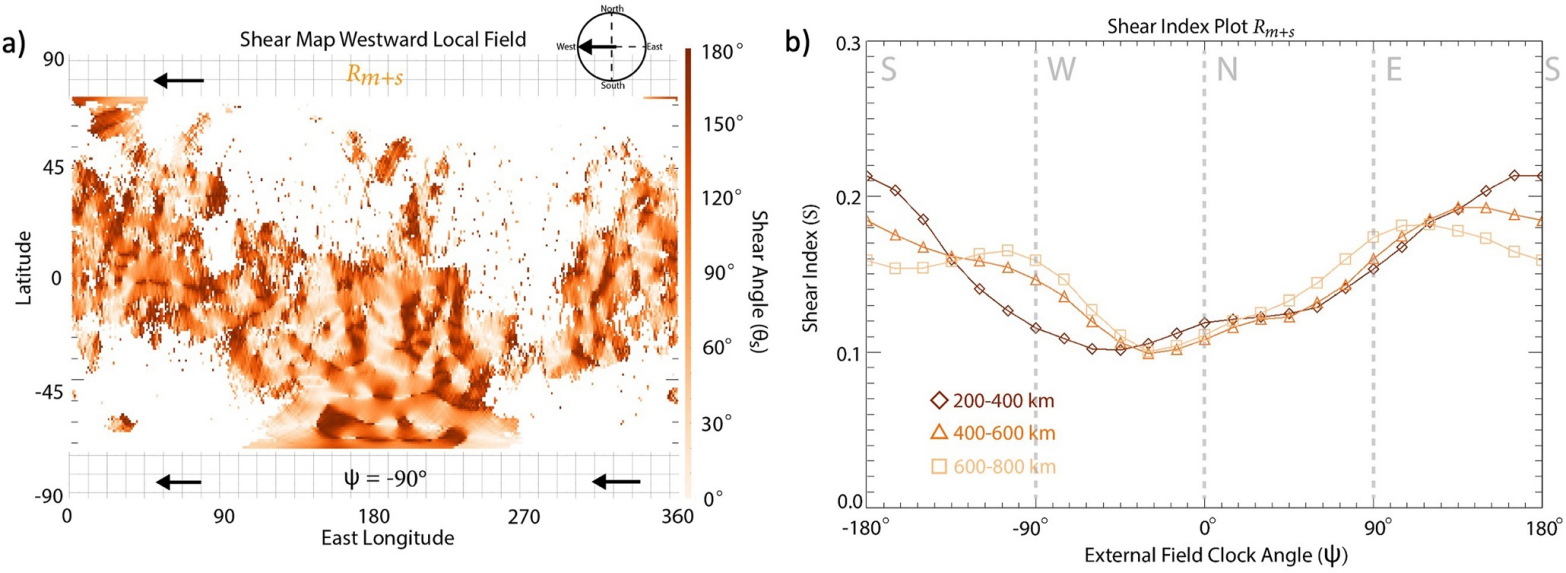
Shear analysis plots for $R_{m+s}$ for the data‐derived crustal field map. (a) Shear map of crustal magnetic fields for a westward draped field orientation measured from 200 to 400 km altitude for $R_{m+s}$: 20 nT < ${|B}_{cf}|$. Shear index plot (b) from crustal magnetic field data measured within three different altitude ranges: 200–400 km (diamonds), 400–600 km (triangles), and 600–800 km (squares).

Based on these results, we combine $R_{\mathrm{mod}}$ and $R_{strong}$ (the purple and green regions of Figure 8a) for the data‐derived crustal field map to define $R_{m+s}$, the crustal field region in which the crustal magnetic field strength exceeds 20 nT at the lowest altitude range (20 nT ${<\;|B}_{cf}|$). Figure 10 shows a shear map and S plot for $R_{m+s}$. The S plot (Figure 10b), shows similar trends in S to those presented earlier for the equatorial (Figure 7c) and moderate (Figure 8c) crustal fields. One such trend is the tendency for the shear index, S, to maximize (S≈0.19–0.21) for southward external field orientations at low‐midaltitude ranges. At the highest altitude range (600–800 km), we still see a preference in S for southward fields (S≈0.15) compared to northward (S≈0.1) fields, but we also see local maxima in S for eastward and westward fields (S≈0.17) as well. These results suggest southward external fields are the most likely to globally affect the magnetosphere and result in magnetic reconnection with the moderate‐to‐strong crustal fields.

This result also relates to the dayside interaction between the moderate‐to‐strong crustal magnetic fields and the IMF in the MSO coordinate system. We note that our results provide a framework for understanding the preferred external field geometry in the magnetosheath that is adjacent to the magnetopause. However, caution must be taken when drawing connections between the upstream IMF orientation in the MSO frame and the local, external magnetic field orientation analyzed in our study because the IMF rotates as it crosses the bow shock and drapes around the planet (Chai et al., 2019; Dubinin et al., 2019; Fang et al., 2018). For this application of our analysis to the IMF‐Mars interaction, we assume that a southward‐directed field corresponds to a − *Z*_MSO_‐draped IMF that is adjacent to the magnetopause and directly interacting with the crustal fields. This implies that a − *Z*_MSO_‐directed IMF located in the magnetosheath, interacting with the crustal fields would create the largest area of high shear with the moderate‐strong crustal magnetic fields on the dayside (Figure 10b). Therefore, we suggest that dayside magnetic reconnection between the draped IMF and the underlying crustal anomalies is most likely to occur when the IMF is oriented in the − *Z*_MSO_ direction. This preference is similar to that at Earth, where − *Z*_GSM_ conditions favor the onset of magnetic reconnection along the terrestrial magnetopause. The − *Z*_MSO_ IMF preference for reconnection at Mars is not obvious due to the nonuniform nature of the crustal anomalies. Our results suggest the orientations of scattered crustal anomalies at Mars demonstrate global preferences despite their variations over small scales.

Next, we interpret this preference for IMF direction to enable magnetic reconnection to provide context for global open topology observations at Mars (Brain et al., 2020; Weber et al., 2020; Xu et al., 2019, 2020). If we assume dayside magnetic reconnection between the crustal anomalies and IMF produces the majority of open magnetic fields, then our study predicts open topology around Mars should be observed most frequently under southward (−Z_MSO_) IMF conditions. Xu et al. (2018) demonstrated that dayside open topology rates increased during the 2017 September interplanetary coronal mass ejection (ICME) impact at Mars. Our study suggests this enhancement in open topology is in part due to enhanced magnetic reconnection rates between the crustal anomalies and the prominent southward IMF associated with ICME impacts. Recent studies using the Mars Global Surveyor (MGS) spacecraft and MAVEN have revealed variations in nightside, open topology measurements with the ± *Y*_MSO_ component of the draped IMF (Brain et al., 2020; Weber et al., 2020). These studies were limited to nightside open magnetic topology rates over magnetic cusps, and thus focused on the interaction between the draped IMF and the radial component of the crustal anomalies on the nightside. In contrast our study focuses on the global trends in dayside magnetic reconnection between an external field and the transverse component of the crustal anomalies. The nightside interaction analyzed in Brain et al. (2020) and Weber et al. (2020) is different than the dayside interaction analyzed in our shear analysis, and this difference likely explains the discrepancy between our results.

The relationship between upstream IMF orientation and global dayside magnetic reconnection also directly impacts the twisted magnetotail. Field line tracings of 3D multispecies magnetohydrodynamic (MHD) simulations (DiBraccio et al., 2018) and magnetic topology estimates using MAVEN particle and field observations (Xu et al., 2020) suggest that the twisted tail is mostly composed of open magnetic field lines. The direction of this twist has been observed to depend heavily on the ± *Y*_MSO_ component of the IMF, but the reasoning behind this dependency is still being explored. When assessing the influence of the strong crustal fields on tail twisting, DiBraccio et al. (2022) found that the degree of twisting varies with the IMF *Y*_MSO_ component, and the largest tail twist of nearly 60° occurs for +*Y*_MSO_ when the strongest crustal sources are located on the nightside. Additionally, DiBraccio et al. (2022) found that the tail twist was at a minimum, of ∼5°–10°, for both ± *Y*_MSO_ IMF conditions when the strong crustal fields were at dusk (18:00 LT). These results further suggest that the twisted tail is a result of a more global IMF‐planetary interaction that is an aggregation of localized interactions between IMF and the strongest crustal fields.

At Earth, the magnetotail is twisted by roughly 10° or less, and is controlled by the ± *Y*_GSM_ component of the IMF (e.g., Cowley, 1981; Kaymaz et al., 1994; Sibeck & Lin, 2014; Sibeck et al., 1985; Xiao et al., 2016). The magnetospheres of Mars and Earth both appear to be most susceptible to dayside magnetic reconnection under − *Z*_MSO/GSM_ IMF conditions. Also, they both exhibit a twisted magnetotail that depends on the ± *Y*_MSO/GSM_ component of the IMF. Despite the differences between the two magnetospheres, a comparison between the properties of twisted magnetotail of Mars and Earth may shed light onto the physical mechanisms responsible. Sibeck and Lin (2014) suggested the twisted terrestrial magnetotail results from open magnetic field lines generated via reconnection on the dayside. As these newly generated open field lines propagate to the nightside, they will be pulled in the direction of the IMF and will be asymmetrically added to the duskside/dawnside lobe of the magnetotail depending on the ± *Y*_GSM_ component of the IMF and the magnetic pole in which the open field is connected. The combined influence of the global dipole field and the open magnetic field lines in the terrestrial tail result in a twisted structure. Our results suggest the same processes that twist the terrestrial magnetotail may also be responsible for the twisted Martian magnetotail. In the Martian case, the open field lines within the magnetotail are not connected to a strong global dipole field but are instead connected to much weaker crustal anomalies. In comparison to the global dipole field at Earth, the crustal anomalies at Mars do not exert as strong an influence on the magnetotail, and the open field lines generated on the dayside therefore produce a more extreme twisted magnetotail on the nightside that varies with the location of the crustal anomalies, as reported by DiBraccio et al. (2022). However, future studies dedicated to connecting the nature of the twisted magnetotail with dayside reconnection between the IMF and the dayside magnetosphere is required to fully understand the processes responsible for shaping the magnetotail of both Earth and Mars.

In this shear analysis investigation, we have presented a tool that assesses the likelihood of magnetic reconnection to occur across localized regions of the Martian crustal fields, and then provided a method for interpreting this on a global scale. The global assessment has suggested that high shear, antiparallel magnetic reconnection between the IMF and moderate‐to‐strong crustal magnetic anomalies on the dayside occur most frequently for −Z_MSO_ IMF conditions. These results hold implications for trends in reconnection‐related phenomena including open topology measurements and the twisted configuration of the Martian magnetotail. Moreover, the shear analysis provides important insights to understanding the complex nature of the solar wind‐Mars interaction as we continue to explore the processes driving its hybrid structure.

## Data Availability

In accordance with the AGU data policy, MAVEN data are publicly available through the Planetary Plasma Interactions Node of the Planetary Data System (https://pds-ppi.igpp.ucla.edu/mission/MAVEN).

## References

[jgra57640-bib-0001] Acuña, M. H. , Connerney, J. E. , Ness, R. P. , Mitchell, D. , Carlson, C. W. , McFadden, J. , et al. (1999). Global distribution of crustal magnetization discovered by the Mars global surveyor Mag/Er experiment. Science, 284(5415), 790–793. 10.1126/science.284.5415.790 10221908

[jgra57640-bib-0002] Beharrell, M. , & Wild, J. (2012). Stationary flux ropes at the southern terminator of Mars. Journal of Geophysical Research, 117, A12212. 10.1029/2012JA017738

[jgra57640-bib-0003] Bowers, C. , Slavin, J. , DiBraccio, G. , Poh, G. , Hara, T. , Xu, S. , & Brain, D. (2021). MAVEN survey of magnetic flux rope properties in the Martian ionosphere: Comparison with three types of formation mechanisms. Geophysical Research Letters, 48, e2021GL093296. 10.1029/2021GL093296

[jgra57640-bib-0004] Brain, D. , Bagenal, F. , Acuna, M. , & Connerney, J. (2003). Martian magnetic morphology: Contributions from the solar wind and crust. Journal of Geophysical Research, 108(A12), 1424. 10.1029/2002JA009482

[jgra57640-bib-0005] Brain, D. , Baker, A. , Briggs, J. , Eastwood, J. , Halekas, J. , & Phan, T. (2010). Episodic detachment of Martian crustal magnetic fields leading to Bulk atmospheric plasma escape. Geophysical Research Letters, 37, L14108. 10.1029/2010GL043916

[jgra57640-bib-0006] Brain, D. , Halekas, J. , Lillis, R. , Mitchell, D. , Lin, R. , & Crider, D. (2005). Variability of the altitude of the Martian Sheath. Geophysical Research Letters, 32, L18203. 10.1029/2005GL023126

[jgra57640-bib-0007] Brain, D. , Lillis, R. , Mitchell, D. , Halekas, J. , & Lin, R. (2007). Electron pitch angle distributions as Indicators of magnetic field topology near Mars. Journal of Geophysical Research, 112, A09201. 10.1029/2007JA012435

[jgra57640-bib-0008] Brain, D. , Weber, T. , Xu, S. , Mitchell, D. , Lillis, R. , Halekas, J. , et al. (2020). Variations in nightside magnetic field topology at Mars. Geophysical Research Letters, 47, e2020GL088921. 10.1029/2020GL088921

[jgra57640-bib-0009] Briggs, J. , Brain, D. , Cartwright, M. , Eastwood, J. , & Halekas, J. (2011). A statistical study of flux ropes in the Martian magnetosphere. Planetary and Space Science, 59(13), 1498–1505. 10.1016/j.pss.2011.06.010

[jgra57640-bib-0010] Chai, L. , Wan, W. , Wei, Y. , Zhang, T. , Exner, W. , Fraenz, M. , et al. (2019). The induced global looping magnetic field on Mars. The Astrophysical Journal Letters, 871(2), L27. 10.3847/2041-8213/aaff6e

[jgra57640-bib-0011] Connerney, J. , Espley, J. , Lawton, P. , Murphy, S. , Odom, J. , Oliversen, R. , & Sheppard, D. (2015). The MAVEN magnetic field investigation. Space Science Reviews, 195(1–4), 257–291. 10.1007/s11214-015-0169-4

[jgra57640-bib-0012] Cooling, B. , Owen, C. , & Schwartz, S. (2001). Role of the magnetosheath flow in determining the motion of open flux tubes. Journal of Geophysical Research, 106(A9), 18763–18775. 10.1029/2000JA000455

[jgra57640-bib-0013] Cowley, S. (1981). Magnetospheric asymmetries associated with the Y‐component of the IMF. Planetary and Space Science, 29(1), 79–96. 10.1016/0032-0633(81)90141-0

[jgra57640-bib-0014] Cravens, T. , Fowler, C. , Brain, D. , Rahmati, A. , Xu, S. , Ledvina, S. , et al. (2020). Magnetic reconnection in the ionosphere of Mars: The role of collisions. Journal of Geophysical Research‐Space Physics, 125, e2020JA028036. 10.1029/2020JA028036

[jgra57640-bib-0015] Cravens, T. , Rahmati, A. , Fox, J. L. , Lillis, R. , Bougher, S. , Luhmann, J. , et al. (2017). Hot oxygen escape from Mars: Simple scaling with solar EUV irradiance. Journal of Geophysical Research: Space Physics, 122, 1102–1116. 10.1002/2016JA023461

[jgra57640-bib-0016] Crooker, N. (1979). Dayside merging and cusp geometry. Journal of Geophysical Research, 84(NA3), 951–959. 10.1029/JA084iA03p00951

[jgra57640-bib-0017] de Oliveira, I. , Franz, M. , Echer, E. , & Franco, A. (2021). Advection of Martian crustal magnetic fields by ionospheric plasma flow observed by the MAVEN spacecraft. Journal of Geophysical Research‐Space Physics, 126, e2021JA029242. 10.1029/2021JA029242

[jgra57640-bib-0018] Desroche, M. , Bagenal, F. , Delamere, P. , & Erkaev, N. (2012). Conditions at the expanded Jovian magnetopause and implications for the solar wind interaction. Journal of Geophysical Research, 117. A07202. 10.1029/2012JA017621

[jgra57640-bib-0019] DiBraccio, G. , Luhmann, J. G. , Curry, S. M. , Espley, J. R. , Xu, S. , Mitchell, D. L. , et al. (2018). The twisted configuration of the Martian magnetotail: MAVEN observations. Geophysical Research Letters, 45, 4559–4568. 10.1029/2018GL077251

[jgra57640-bib-0020] DiBraccio, G. , Romanelli, N. , Bowers, C. F. , Gruesbeck, J. R. , Halekas, J. S. , Ruhunusiri, S. , et al. (2022). A statistical investigation of Factors influencing the magnetotail twist at Mars. Geophysical Research Letters, 49, e2022GL098007. 10.1029/2022GL098007 PMC9286686 35865912

[jgra57640-bib-0021] Dubinin, E. , Modolo, R. , Fraenz, M. , Paetzold, M. , Woch, J. , Chai, L. , et al. (2019). The induced magnetosphere of Mars: Asymmetrical topology of the magnetic field lines. Geophysical Research Letters, 46, 12722–12730. 10.1029/2019GL084387

[jgra57640-bib-0022] Dubinin, E. , Modolo, R. , Fraenz, M. , Woch, J. , Duru, F. , Akalin, F. , et al. (2008). Structure and dynamics of the solar wind/ionosphere interface on Mars: MEX‐ASPERA‐3 and MEX‐MARSIS observations. Geophysical Research Letters, 35, L11103. 10.1029/2008GL033730

[jgra57640-bib-0023] Dungey, J. (1963). Interactions of solar plasma with the geomagnetic field. Planetary and Space Science, 10, 233–237. 10.1016/0032-0633(63)90020-5

[jgra57640-bib-0024] Dungey, J. W. (1961). Interplanetary magnetic field and the Auroral Zones. Physical Review Letters, 6(2), 47–48. 10.1103/PhysRevLett.6.47

[jgra57640-bib-0025] Fang, X. , Ma, Y. , Luhmann, J. , Dong, Y. , Brain, D. , Hurley, D. , et al. (2018). The morphology of the solar wind magnetic field draping on the dayside of Mars and its variability. Geophysical Research Letters, 45, 3356–3365. 10.1002/2018GL077230

[jgra57640-bib-0026] Fuselier, S. , Kletzing, C. A. , Petrinec, S. M. , Trattner, K. J. , George, D. , Bounds, S. R. , et al. (2022). Multiple reconnection X‐lines at the magnetopause and overlapping cusp ion Injections. Journal of Geophysical Research: Space Physics, 127, e2022JA030354. 10.1029/2022JA030354

[jgra57640-bib-0027] Fuselier, S. , Petrinec, S. , Sawyer, R. , Mukherjee, J. , & Masters, A. (2020). Suppression of magnetic reconnection at Saturn's low‐latitude magnetopause. Journal of Geophysical Research: Space Physics, 125, e2020JA027895. 10.1029/2020JA027895

[jgra57640-bib-0028] Fuselier, S. A. , Trattner, K. J. , & Petrinec, S. M. (2011). Antiparallel and component reconnection at the dayside magnetopause. Journal of Geophysical Research, 116, A10227. 10.1029/2011JA016888 PMC5014232 27656333

[jgra57640-bib-0029] Fuselier, S. , Vines, S. K. , Burch, J. L. , Petrinec, S. M. , Trattner, K. J. , Cassak, P. A. , et al. (2017). Large‐scale characteristics of reconnection diffusion regions and associated magnetopause crossings observed by MMS. Journal of Geophysical Research: Space Physics, 122, 5466–5486. 10.1002/2017JA024024

[jgra57640-bib-0030] Hara, T. , Seki, K. , Hasegawa, H. , Brain, D. , Matsunaga, K. , Saito, M. , & Shiota, D. (2014). Formation processes ff flux ropes downstream from Martian crustal magnetic fields inferred from Grad‐Shafranov reconstruction. Journal of Geophysical Research: Space Physics, 119, 7947–7962. 10.1002/2014JA019943

[jgra57640-bib-0031] Harada, Y. , Halekas, J. S. , DiBraccio, G. A. , Xu, S. , Espley, J. , Mcfadden, J. P. , et al. (2018). Magnetic reconnection on dayside crustal magnetic fields at Mars: MAVEN observations. Geophysical Research Letters, 45, 4550–4558. 10.1002/2018GL077281

[jgra57640-bib-0032] Harada, Y. , Halekas, J. , Xu, S. , DiBraccio, G. , Ruhunusiri, S. , Hara, T. , et al. (2020). Ion jets within current sheets in the Martian magnetosphere. Journal of Geophysical Research‐Space Physics, 125, e2020JA028576. 10.1029/2020JA028576

[jgra57640-bib-0033] Jakosky, B. , Lin, R. P. , Grebowsky, J. M. , Luhmann, J. G. , Mitchell, D. F. , Beutelschies, G. , et al. (2015). The Mars atmosphere and volatile evolution (MAVEN) mission. Space Science Reviews, 195(1–4), 3–48. 10.1007/s11214-015-0139-x

[jgra57640-bib-0034] Kaweeyanun, N. , Masters, A. , & Jia, X. (2020). Favorable conditions for magnetic reconnection at Ganymede's upstream magnetopause. Geophysical Research Letters, 47, e2019GL086228. 10.1029/2019GL086228

[jgra57640-bib-0035] Kaymaz, Z. , Siscoe, G. , Luhmann, J. , Lepping, R. , & Russell, C. (1994). Interplanetary magnetic‐field Control of magnetotail magnetic‐field geometry—Imp‐8 observations. Journal of Geophysical Research, 99(A6), 11113–11126. 10.1029/94JA00300

[jgra57640-bib-0036] Langlais, B. , Thebault, E. , Houliez, A. , Purucker, M. , & Lillis, R. (2019). A new model of the crustal magnetic field of Mars using MGS and MAVEN. Journal of Geophysical Research: Planets, 124, 1542–1569. 10.1029/2018JE005854 35096494 PMC8793354

[jgra57640-bib-0037] Lillis, R. , Fillingim, M. , & Brain, D. (2011). Three‐dimensional structure of the Martian nightside ionosphere: Predicted rates of impact ionization from Mars global surveyor magnetometer and electron reflectometer measurements of precipitating electrons. Journal of Geophysical Research, 116, A12317. 10.1029/2011JA016982

[jgra57640-bib-0038] Luhmann, J. , Ledvina, S. , Russell, C. , & BlancoCano, X. (2004). Induced magnetospheres. Comparative Magnetospheres, 33(11), 1905–1912. 10.1016/j.asr.2003.03.031

[jgra57640-bib-0039] Ma, Y. , Nagy, A. , Hansen, K. , DeZeeuw, D. , Gombosi, T. , & Powell, K. (2002). Three‐dimensional multispecies MHD studies of the solar wind interaction with Mars in the presence of crustal fields. Journal of Geophysical Research, 107(A10), 1282. 10.1029/2002JA009293

[jgra57640-bib-0040] Masters, A. (2014). Magnetic reconnection at Uranus' magnetopause. Journal of Geophysical Research: Space Physics, 119, 5520–5538. 10.1002/2014JA020077

[jgra57640-bib-0041] Masters, A. (2015). Magnetic reconnection at Neptune's magnetopause. Journal of Geophysical Research: Space Physics, 120, 479–493. 10.1002/2014JA020744 PMC5014232 27656333

[jgra57640-bib-0042] McFadden, J. , Kortmann, O. , Curtis, D. , Dalton, G. , Johnson, G. , Abiad, R. , et al. (2015). MAVEN SupraThermal and thermal ion Compostion (STATIC) instrument. Space Science Reviews, 195(1–4), 199–256. 10.1007/s11214-015-0175-6

[jgra57640-bib-0043] Mitchell, D. , Mazelle, C. , Sauvaud, J. A. , Thocaven, J. J. , Rouzaud, J. , Fedorov, A. , et al. (2016). The MAVEN solar wind electron analyzer. Space Science Reviews, 200(1–4), 495–528. 10.1007/s11214-015-0232-1

[jgra57640-bib-0044] Paschmann, G. , Oieroset, M. , & Phan, T. (2013). Situ observations of reconnection in space. Space Science Reviews, 178(2–4), 385–417. 10.1007/s11214-012-9957-2

[jgra57640-bib-0045] Paschmann, G. , Sonnerup, B. , Papamastorakis, I. , Sckopke, N. , Haerendel, G. , Bame, S. , et al. (1979). Plasma acceleration at the Earth’s magnetopause—Evidence for reconnection. Nature, 282(5736), 243–246. 10.1038/282243a0

[jgra57640-bib-0046] Petrinec, S. , Burch, J. L. , Chandler, M. , Farrugia, C. J. , Fuselier, S. A. , Giles, B. L. , et al. (2020). Characteristics of minor ions and electrons in flux transfer events observed by the magnetospheric Multiscale mission. Journal of Geophysical Research: Space Physics, 125, e2020JA027778. 10.1029/2020JA027778 PMC7507212 32999806

[jgra57640-bib-0047] Petrinec, S. , Burch, J. L. , Fuselier, S. A. , Gomez, R. G. , Lewis, W. , Trattner, K. J. , et al. (2016). Comparison of magnetospheric multiscale ion jet signatures with predicted reconnection site locations at the magnetopause. Geophysical Research Letters, 43, 5997–6004. 10.1002/2016GL069626

[jgra57640-bib-0048] Sibeck, D. , & Lin, R. (2014). Size and shape of the distant magnetotail. Journal of Geophysical Research: Space Physics, 119, 1028–1043. 10.1002/2013JA019471

[jgra57640-bib-0049] Sibeck, D. , Siscoe, G. , Slavin, I. , Smith, E. , Tsurutani, B. , & Lepping, R. R. (1985). The distant magnetotails response to a strong interplanetary magnetic‐field by—Twisting, flattening, and field line bending. Journal of Geophysical Research, 90(NA5), 4011–4019. 10.1029/JA090iA05p04011

[jgra57640-bib-0050] Slavin, J. , Anderson, B. J. , Baker, D. N. , Benna, M. , Boardsen, S. A. , Gloeckler, G. , et al. (2010). MESSENGER observations of extreme loading and unloading of Mercury's magnetic tail. Science, 329(5992), 665–668. 10.1126/science.1188067 20647422

[jgra57640-bib-0051] Slavin, J. , Imber, S. M. , Boardsen, S. A. , DiBraccio, G. A. , Sundberg, T. , Sarantos, M. , et al. (2012). MESSENGER observations of a flux‐transfer‐event shower at Mercury. Journal of Geophysical Research, 117, A00M06. 10.1029/2012JA017926

[jgra57640-bib-0052] Sonnerup, B. U. Ö. , & Scheible, M. (1998). Minimum and maximum variance analysis. In Analysis methods for multi‐spacecraft data. (ISSI Sci. Rep. Ser. SR‐001). ISSI/ESA.

[jgra57640-bib-0053] Trattner, K. , Mulcock, J. , Petrinec, S. , & Fuselier, S. (2007a). Location of the reconnection line at the magnetopause during southward IMF conditions. Geophysical Research Letters, 34, L03108. 10.1029/2006GL028397

[jgra57640-bib-0054] Trattner, K. , Mulcock, J. , Petrinec, S. , & Fuselier, S. (2007b). Probing the boundary between antiparallel and component reconnection during southward interplanetary magnetic field conditions. Journal of Geophysical Research, 112, A08210. 10.1029/2007JA012270

[jgra57640-bib-0055] Trattner, K. , Petrinec, S. , Fuselier, S. , & Phan, T. (2012). The location of reconnection at the magnetopause: Testing the maximum magnetic shear model with Themis observations. Journal of Geophysical Research, 117, A01201. 10.1029/2011JA016959

[jgra57640-bib-0056] Trotignon, J. , Mazelle, C. , Bertucci, C. , & Acuna, M. (2006). Martian shock and magnetic Pile‐up boundary positions and shapes determined from the Phobos 2 and Mars global surveyor data sets. Planetary and Space Science, 54(4), 357–369. 10.1016/j.pss.2006.01.003

[jgra57640-bib-0057] Tsyganenko, N. , & Stern, D. (1996). Modeling the global magnetic field of the large‐scale Birkeland current systems. Journal of Geophysical Research, 101(A12), 27187–27198. 10.1029/96JA02735

[jgra57640-bib-0058] Wang, J. , Yu, J. , Xu, X. , Cui, J. , Cao, J. , Ye, Y. , et al. (2021). MAVEN observations of magnetic reconnection at Martian induced magnetopause. Geophysical Research Letters, 48, e2021GL095426. 10.1029/2021GL095426

[jgra57640-bib-0059] Weber, T. , Brain, D. , Mitchell, D. , Xu, S. , Connerney, J. , & Halekas, J. (2017). Characterization of low‐altitude nightside Martian magnetic topology using electron pitch angle distributions. Journal of Geophysical Research: Space Physics, 122, 9777–9789. 10.1002/2017JA024491

[jgra57640-bib-0060] Weber, T. , Brain, D. , Xu, S. , Mitchell, D. , Espley, J. , Halekas, J. , et al. (2020). The influence of interplanetary magnetic field direction on Martian crustal magnetic field topology. Geophysical Research Letters, 47, e2020GL087757. 10.1029/2020GL087757

[jgra57640-bib-0061] Weber, T. D. (2020). The role of crustal magnetic fields in atmospheric escape from Mars (Order No. 28149912) (Dissertations & Theses Global, 2474837751). Retrieved from https://www.proquest.com/dissertations-theses/role-crustal-magnetic-fields-atmospheric-escape/docview/2474837751/se-2?accountid=14667

[jgra57640-bib-0062] Xiao, S. , Zhang, T. , Ge, Y. , Wang, G. , Baumjohann, W. , & Nakamura, R. (2016). A statistical study on the shape and position of the magnetotail neutral sheet. Annales Geophysicae, 34(2), 303–311. 10.5194/angeo-34-303-2016

[jgra57640-bib-0063] Xu, S. , Fang, X. , Mitchell, D. L. , Ma, Y. , Luhmann, J. G. , DiBraccio, G. A. , et al. (2018). Investigation of Martian magnetic topology response to 2017 September ICME. Geophysical Research Letters, 45, 7337–7346. 10.1029/2018GL077708

[jgra57640-bib-0064] Xu, S. , Liemohn, M. , & Mitchell, D. (2014). Solar wind electron precipitation into the dayside Martian upper atmosphere through the cusps of strong crustal fields. Journal of Geophysical Research: Space Physics, 119, 10100–10115. 10.1002/2014JA020363

[jgra57640-bib-0065] Xu, S. , Mitchell, D. , Liemohn, M. , Fang, X. , Ma, Y. , Luhmann, J. , et al. (2017). Martian low‐altitude magnetic topology Deduced from Maven/SWEA observations. Journal of Geophysical Research: Space Physics, 122, 1831–1852. 10.1002/2016JA023467

[jgra57640-bib-0066] Xu, S. , Mitchell, D. L. , Weber, T. , Brain, D. A. , Luhmann, J. G. , Dong, C. , et al. (2020). Characterizing Mars's magnetotail topology with respect to the upstream interplanetary magnetic fields. Journal of Geophysical Research: Space Physics, 125, e2019JA027755. 10.1029/2019JA027755

[jgra57640-bib-0067] Xu, S. , Weber, T. , Mitchell, D. , Brain, D. , Mazelle, C. , DiBraccio, G. , & Espley, J. (2019). A technique to infer magnetic topology at Mars and its application to the terminator region. Journal of Geophysical Research: Space Physics, 124, 1823–1842. 10.1029/2018JA026366

